# Alterations in the Colonic Microbiota of Pigs Associated with Feeding Distillers Dried Grains with Solubles

**DOI:** 10.1371/journal.pone.0141337

**Published:** 2015-11-10

**Authors:** Eric R. Burrough, Bailey L. Arruda, John F. Patience, Paul J. Plummer

**Affiliations:** 1 Department of Veterinary Diagnostic and Production Animal Medicine, College of Veterinary Medicine, Iowa State University, Ames, IA, United States of America; 2 Department of Animal Science, Iowa State University, Ames, IA, United States of America; 3 Department of Veterinary Microbiology and Preventative Medicine, College of Veterinary Medicine, Iowa State University, Ames, IA, United States of America; University Paris South, FRANCE

## Abstract

In an effort to reduce feed costs, many pork producers have increased their use of coproducts of biofuel production in commercial pig diets, including increased feeding of distiller’s dried grains with solubles (DDGS). The inclusion of DDGS increases the insoluble fiber content in the ration, which has the potential to impact the colonic microbiota considerably as the large intestine contains a dynamic microenvironment with tremendous interplay between microorganisms. Any alteration to the physical or chemical properties of the colonic contents has the potential to impact the resident bacterial population and potentially favor or inhibit the establishment of pathogenic species. In the present study, colonic contents collected at necropsy from pigs fed either 30% or no DDGS were analyzed to examine the relative abundance of bacterial taxa associated with feeding this ingredient. No difference in alpha diversity (richness) was detected between diet groups. However, the beta diversity was significantly different between groups with feeding of DDGS being associated with a decreased Firmicutes:Bacteriodetes ratio (*P* = .004) and a significantly lower abundance of *Lactobacillus* spp. (*P* = .016). Predictive functional profiling of the microbiota revealed more predicted genes associated with carbohydrate metabolism, protein digestion, and degradation of glycans in the microbiota of pigs fed DDGS. Taken together, these findings confirm that alterations in dietary insoluble fiber significantly alter the colonic microbial profile of pigs and suggest the resultant microbiome may predispose to the development of colitis.

## Introduction

The diversion of an increasing portion of the U.S. corn crop to ethanol production has resulted in both an increase in feed costs for pork producers associated with corn-based diets and also an increase in availability of ethanol coproducts for use in animal feeds. In order to reduce production costs, many pork producers have increased their use of such coproducts in commercial pig diets, including distiller’s dried grains with solubles (DDGS). Compared to corn, DDGS have considerably more fiber which is predominantly insoluble nonstarch polysaccharides of low fermentability including arabinoxylans and cellulose, as well as lignin [1, 2]. On average, DDGS contain approximately 35% insoluble dietary fiber [3] and it is expected that when pigs are fed diets containing DDGS, a large amount of this insoluble fiber will pass through to the colon. While it is known that hindgut fermentation of total dietary fiber from DDGS is limited to about 25% in pigs [4], the impact of this fiber on the colonic microbiota and associated metagenome has not been well-described.

The large intestine supports a dynamic microenvironment that varies with age and environmental factors [5]. Any alteration to the physical or chemical properties of the colonic contents has the potential to impact the resident bacterial population and potentially favor or inhibit the establishment of pathogenic species. While the addition of DDGS has been shown to affect colonic fermentation rates and apparent total tract digestibility relative to corn alone [4], the precise impact of such a diet change on the colonic microbiota has not been fully characterized. In the present study, colonic contents were collected at necropsy from pigs fed either 30% or no DDGS and analyzed to investigate the relative abundance of bacteria associated with feeding each diet. The *a priori* hypothesis of this study was that feeding 30% DDGS to pigs would significantly alter the colonic microbiota and associated metagenome relative to those not consuming DDGS and that pigs consuming DDGS would develop a microbial profile with features that may predispose pigs to the development of colitis.

## Materials and Methods

### Colonic content samples

All colonic content samples utilized in the present study were collected from twenty crossbred commercial pigs used as uninoculated controls in a previous experiment where pigs were fed one of two diets (n = 10 per diet group) containing either no (Diet 1) or 30% (Diet 2) DDGS for 5 weeks [6]. The diets were prepared in mash form at the Iowa State University Swine Nutrition Farm and the specifics for each formulation are presented in Table 1. All animal procedures were approved by the Institutional Animal Care and Use Committee of Iowa State University (Log Number: 1-12-7283). Contents were collected from the apex of the spiral colon at necropsy which was performed at the conclusion of the previous experiment when pigs were approximately 9-weeks-old. Animals were euthanized by intravenous barbiturate overdose and the spiral colon was exteriorized within approximately 5 minutes of the time of death. An incision was made at the apex of the spiral colon using a separate set of disinfected instruments for each pig. The luminal contents were then collected into individual sterile 2.0 ml cryogenic vials (Corning Inc., Corning, NY) and were flash frozen in liquid nitrogen and retained at -80°C until processing.

**Table 1:** Ingredient and nutrient composition of experimental diets.

Component	Diet 1 (%)	Diet 2 (%)
*Ingredients*		
Corn, yellow dent	61.13	34.55
Corn DDGS	0	30.00
Soybean meal	20.00	17.50
Fish meal, Menhaden Select	5.66	5.06
Whey, dried	10.00	10.00
l-Lysine HCl	0.31	0.31
dl-Methionine	0.12	0.02
l-Threonine	0.12	0.03
l-Tryptophan	0.02	0.01
Monocalcium phosphate	0.48	0.10
Limestone	0.43	0.70
Salt	0.35	0.35
Vitamin premix^c^	0.23	0.23
Trace mineral premix^d^	0.15	0.15
Soybean oil	1.00	1.00
ME, Mcal/kg	1.54	1.54
Crude Protein, %	19.6	23.9
SID Lysine, %	1.23	1.23
SID Threonine, %	0.76	0.76
SID TSAA, %	0.71	0.71
SID Tryptophan, %	0.21	0.21
ADF, %^a^	3.0	5.7
NDF, %^b^	7.9	14.3
Crude Fat, %	4.6	6.4
Calcium, %	0.70	0.71
Phosphorus, %	0.65	0.67
Sodium, %	0.27	0.33
Chloride, %	0.42	0.46

^a^ADF = Acid detergent fiber

^b^NDF = Neutral detergent fiber

^c^Provided per kg of diet: 7,000 IU vitamin A, 800 IU vitamin D3, 57 IU vitamin E, 3.4, menadione, 13 mg riboflavin, 64 mg niacin, 31 mg pantothenic acid, and 57 μg vitamin B12

^d^Provided per kg of diet: 165 mg Zn as ZnSO4, 165 mg Fe as FeSO4, 39 mg Mn as MnSO4, 17 mg Cu as CuSO4, 0.3 mg I as Ca(IO3)2 and 0.3 mg Se as Na2SeO3

### DNA Purification

Colonic contents were processed for DNA extraction using the Qiagen DNA Stool MiniKit following the manufacturer’s recommendations. Following DNA purification, samples were screened for DNA concentration and purity using a Nanodrop DNA Flouremeter and the Qubit fluorometer (Life Technologies, Grand Island, NY) and DNA was stored at -80°C prior to downstream processing.

### 16S sequencing

The twenty extracted colonic content samples were submitted to Argonne National Laboratory—Institute for Genomics and Systems Biology Next Generation Sequencing Core (http://ngs.igsb.anl.gov/) to be utilized for metagenomic analysis using amplification of the V3-V4 hypervariable region of the bacterial 16S rRNA gene. All samples were processed by the routine methodology of the core laboratory. Briefly, amplicons were synthesized using a universal 16S forward primer (515F) and 20 unique Golay barcoded reverse primers (806R) as described [7]. Appropriate positive and negative controls were included by the sequencing facility. Sample library DNA concentrations were quantified and samples were pooled with equal amounts of DNA. The pooled libraries were cleaned up with the MO-BIO UltraClean PCR Clean-Up Kit and the concentration was then diluted to 2 nM. A single flow cell lane containing 100 samples (the 20 samples of this report and 80 additional samples) of 300-bp paired end sequences was run on the Illumina MiSeq.

### Metagenomic data analysis

Forward and reverse reads from the paired end sequencing were first merged using the fastq.join script. Qiime 1.8 was then used for additional data analysis. De-multiplexing and quality filtering were then performed using the split_libraries_fastq.py script. The pick_reference_otus_through_otu_table.py script was used for operational taxonomic unit (OTU) calling and taxonomic assignment was performed based on the greengenes database [8]. Comparisons of specific OTUs within groups were made at the phylum, order, and genus level and only those OTUs detected in at least 25% of samples were included in the analysis. The linear discriminant analysis effect size (LEfSe) method [9] was used to assess the biological effect size of observed differences between groups, and predictive functional profiling of the microbiota was performed using the phylogenetic investigation of communities by reconstruction of unobserved states (PICRUSt) approach [10]. Predicted bacterial gene counts in each sample were compared for thirty-eight preselected Kyoto Encyclopedia of Genes and Genomes (KEGG) pathways involving biosynthesis and metabolism of various substrates.

### Statistical analyses

Statistical output was generated by Qiime 1.8. Prior to calculations, all libraries were adjusted to 47,000 reads to avoid potential interpretation errors due to variable sampling depth. Alpha diversity (chao1) was compared using a nonparametric two sample t-test with 999 Monte Carlo permutations. Beta diversity (Bray-Curtis dissimilarity) was compared using a two-sided student’s two-sample t-test with Bonferroni correction. The frequency of detection (group significance) of specific OTU calls within groups was compared using a Kruskal-Wallis nonparametric analysis of variance with Bonferroni correction where appropriate. Firmicutes:Bacteroidetes ratios were calculated based upon the relative abundance percentages reported in Qiime and were compared using a two-sided student’s two-sample t-test. Statistical significance was defined as *P* < .05.

## Results

### Metagenomic data analysis

**Fig 1:**
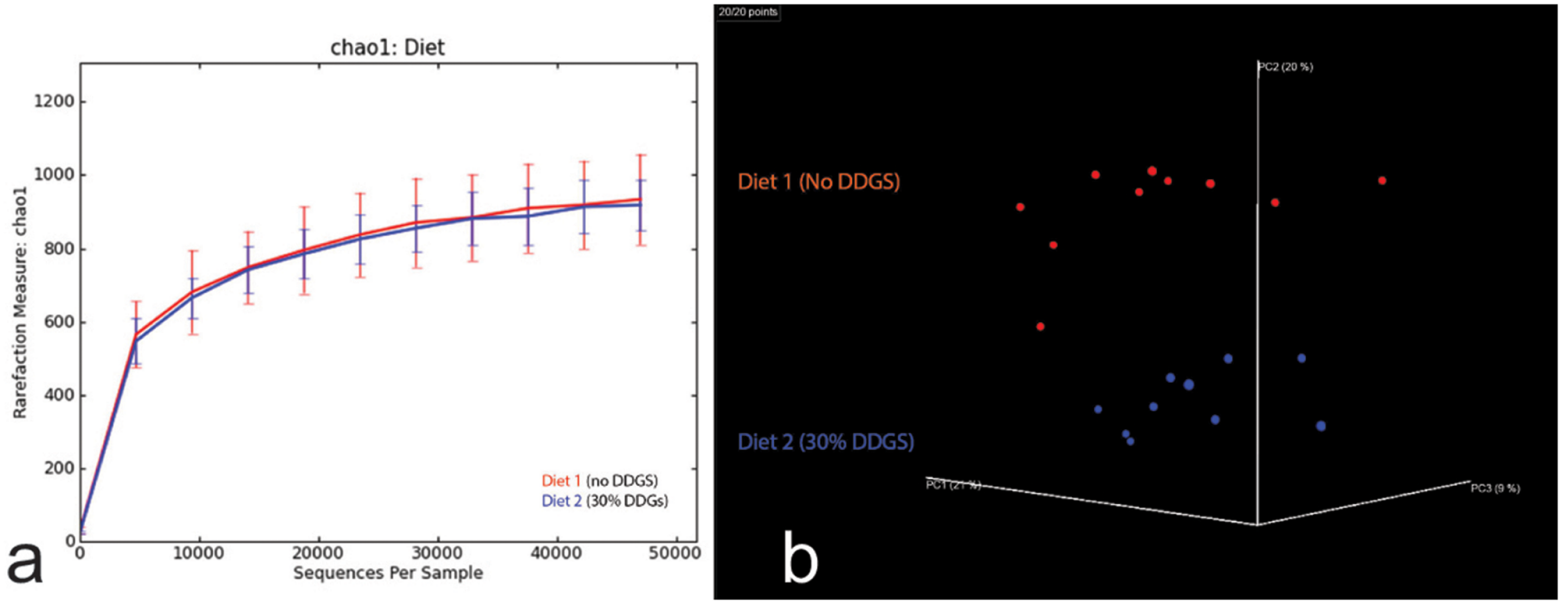
Feeding increased insoluble fiber to pigs alters beta but not alpha diversity in the colonic microbiota. (a) Rarefaction curves comparing alpha diversity (chao1) of colonic microbiota samples from pigs fed either Diet 1 (no DDGS) or Diet 2 (30% DDGS) and revealing no significant differences in richness (*P* = .736; n = 10 samples per diet group). (b) PCoA plot demonstrating significant beta diversity (Bray-Curtis dissimilarity) between diet groups (*P* < .001).

No difference in richness (chao1) was detected between groups (*P* = .736; Fig 1a); however, beta diversity was significantly different revealing clear clustering on a principle coordinates plot of the microbial profiles associated with different diet groups (*P* < .001; Fig 1b).

**Fig 2:**
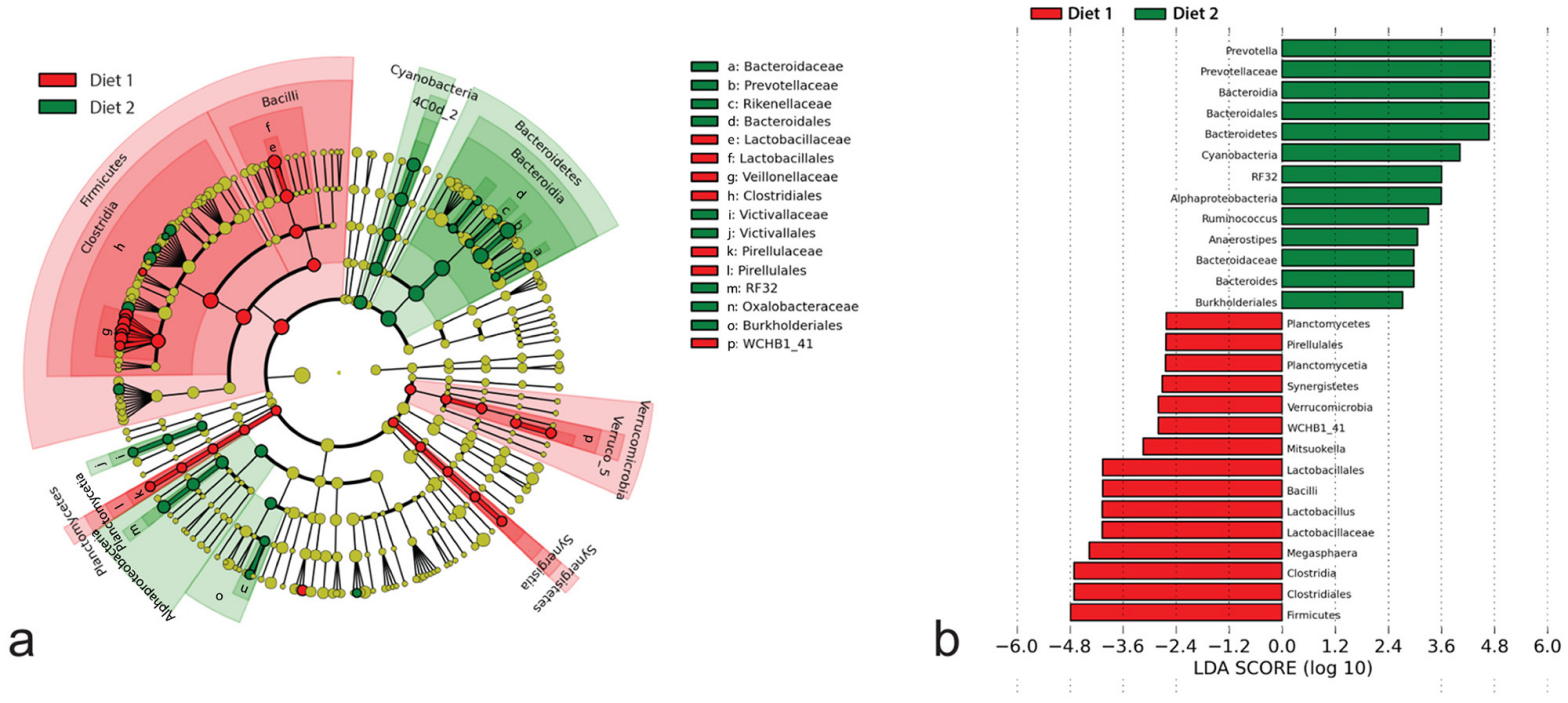
Linear discriminant analysis reveals predicted biological effect sizes of differential taxa in microbiota samples from pigs fed different amounts of insoluble fiber. (a) Cladogram revealing statistically and biologically consistent differences in detected taxa between colonic content samples from pigs fed no DDGS (Diet 1) or 30% DDGS (Diet 2) according to LEfSe. Differences are represented by the color of the diet group in which specific taxa were most abundant (Red = Diet 1, Green = Diet 2) and the diameter of each circle is proportional to the relative abundance. At the phylum level, Firmicutes were more abundant in samples from pigs fed Diet 1 whereas Bacteroidetes were more abundant in samples from pigs fed Diet 2. (b) Histogram of linear discriminant analysis (LDA) scores computed by LEfSe revealing differentially abundant taxa in the microbiota of the two diet groups including overabundance of *Prevotella* spp. and depletion of *Lactobacillus* spp. in pigs fed 30% DDGS (Diet 2).

**Fig 3:**
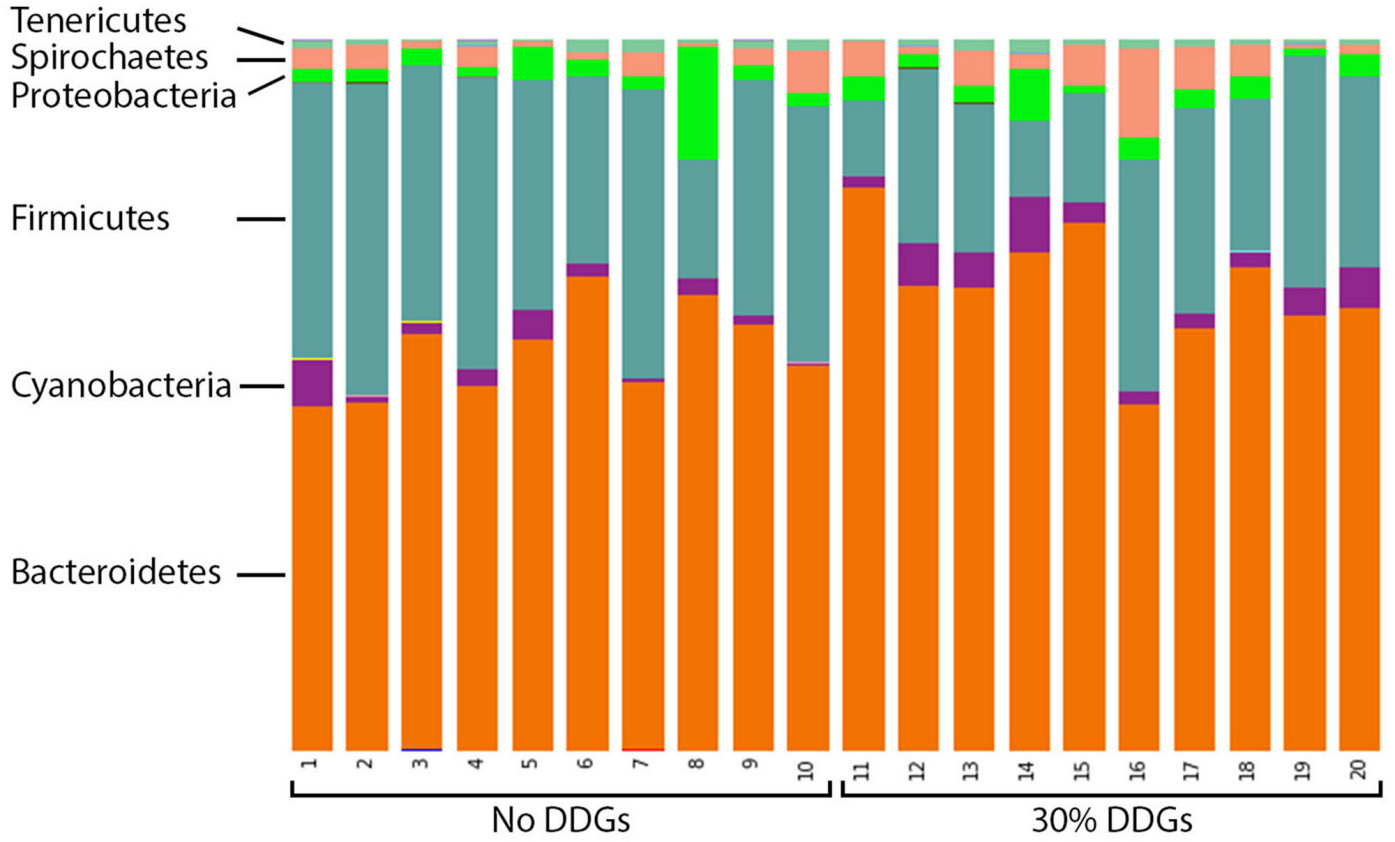
Feeding increased insoluble fiber was associated with shifts in the relative abundance of major phyla in microbiota samples. Stacked bar charts representing proportional abundance of major phyla in the colonic microbiota of twenty 9-week-old pigs fed either no DDGS or 30% DDGS for 5 weeks. Firmicutes:Bacteroidetes ratios were significantly lower in pigs fed 30% DDGS (*P* = .004).

At the phylum level, Planctomycetes and Verrucomicrobia were detected more frequently in samples from pigs fed Diet 1 and these differences were statistically significant (*P* = .004 and *P* = .010, respectively); however, the predicted biological effect size of these differences was relatively low compared to other detected differential features in the linear discriminant analysis (Fig 2a). Bacteroidetes was the dominant phylum detected in all samples regardless of diet fed and ranged from 48.2% to 70.0% of reads detected (Fig 3). Firmicutes were generally more abundant in pigs fed Diet 1 and Bacteroidetes accounted for a higher relative percentage of reads in pigs fed Diet 2 (Fig 3). Accordingly, the Firmicutes:Bacteroidetes ratios were significantly lower in pigs fed Diet 2 (mean 0.361 ± 0.171) relative to pigs fed Diet 1 (mean 0.634 ± 0.201) (*P* = .004). Additionally, Firmicutes was associated with the highest LDA score and was significantly differential for samples from pigs fed Diet 1 while Bacteroidetes was highly differential for samples from Diet 2 pigs (Fig 2b). Other differential features at this level included Synergistetes for samples from Diet 1 pigs and Cyanobacteria for samples from Diet 2 pigs. While Spirochaetes were detected with a higher relative abundance in pigs fed Diet 2 (Fig 3) this difference was not statistically significant or differential by LEfSe.

At the order level, Pirellulales of the Planctomycetes and WCHB1-41 of the Verrucomicrobia were significantly more abundant in colonic content samples from pigs fed Diet 1 (*P* = .007 and *P* = .018, respectively) while RF32 of the Alphaproteobacteria was significantly more abundant in the samples from Diet 2 pigs (*P* = .032). Differential features detected at the order level in LEfSe are shown in Fig 2.

At the genus level, *Lactobacillus* spp., a genus of Pirellulaceae, and *Mitsuokella* spp. were significantly more abundant in the microbiota of pigs fed Diet 1 (*P* = 0.016, *P* = 0.021, and *P* = 0.021, respectively), whereas a genus of Rikenellaceae and *Anaerostipes* spp. were more common in pigs fed Diet 2 (*P* = 0.014 and *P* = 0.016, respectively). The predicted biological significance of these detected differences is shown in Fig 2. Interestingly, while the relative abundance of *Megasphaera* spp. was nearly three times greater in samples from Diet 1 pigs this result was not statistically significant; however, this differential abundance was strongly associated with Diet 1 samples in LEfSe (Fig 2b). Similarly, the relative abundance of *Prevotella* spp., while numerically but not statistically different between sample groups (mean 41.4% for Diet 1 and 51.4% for Diet 2), was the most discriminant feature in Diet 2 samples with an LDA score approaching 4.8 (Fig 2b).

Results of the PICRUSt analysis are summarized in Table 2. Significant differences in the mean number of predicted genes were detected in 14 of the 38 selected KEGG pathways and for all 14 pathways the mean of predicted gene counts was greater for pigs fed Diet 2. Detected differences in the microbiota samples for pigs fed Diet 2 included more predicted genes associated with carbohydrate metabolism, lipid and fatty acid biosynthesis, carotenoid biosynthesis, protein digestion, and degradation of glycosaminoglycans and other glycans.

**Table 2:** Predicted gene counts in microbiota samples from pigs fed diets with and without DDGS.

	Gene count (Mean ± SD)		
KEGG Pathways	Diet 1 (n = 10)	Diet 2 (n = 10)	*P* value
Carotenoid biosynthesis	1237.5 ± 777.0	2553.6 ± 1075.9	0.006
Lipoic acid metabolism	9984.6 ± 2483.7	14459.4 ± 4693.0	0.016
Sphingolipid metabolism	72945.5 ± 15288.4	95841.6 ± 22964.2	0.017
Protein digestion and absorption	20731.1 ± 3883.7	27309.8 ± 7152.9	0.020
Other glycan degradation	107511.0 ± 21418.3	139765.0 ± 34901.7	0.023
Carbohydrate metabolism	48561.9 ± 11521.6	63685.7 ± 15564.8	0.024
Glycosphingolipid biosynthesis—globo series	50702.5 ± 9197.0	64706.1 ± 16578.0	0.031
Lipid biosynthesis proteins	244738.0 ± 49480.2	304743.0 ± 65604.9	0.033
Biosynthesis of unsaturated fatty acids	44730.3 ± 8229.9	55710.9 ± 12680.9	0.034
Starch and sucrose metabolism	371276.0 ± 81521.0	463056.0 ± 103179.0	0.041
Carbohydrate digestion and absorption	14489.0 ± 2957.8	18024.2 ± 4130.2	0.041
Fatty acid biosynthesis	172997.0 ± 34464.2	212864.0 ± 46901.6	0.044
Glycosaminoglycan degradation	33165.3 ± 5845.3	42044.6 ± 11623.0	0.045
Glycosphingolipid biosynthesis—ganglio series	29848.7 ± 5101.4	37899.8 ± 10730.7	0.046
Lipid metabolism	53941.8 ± 11290.3	65849.5 ± 14309.3	0.054
Stilbenoid, diarylheptanoid and gingerol biosythesis	1028.6 ± 822.4	1883.4 ± 1036.6	0.056
Fructose and mannose metabolism	331131.0 ± 69075.0	403198.0 ± 91964.9	0.063
Steroid biosynthesis	155.7 ± 89.8	79.2 ± 89.4	0.072
Energy metabolism	391378.0 ± 76974.0	468183.0 ± 104111.0	0.077
Galactose metabolism	246194.0 ± 54831.2	297576.0 ± 68221.6	0.080
Peptidoglycan biosythesis	392506.0 ± 75597.0	466692.0 ± 101670.0	0.081
Glycan biosynthesis and metabolism	20332.7 ± 3701.3	24407.5 ± 5966.9	0.083
Glycine, serine and threonine metabolism	352694.0 ± 70031.4	417043.0 ± 93456.0	0.099
Glycerophospholipid metabolism	199089.0 ± 42235.2	234623.0 ± 51162.7	0.108
Folate biosynthesis	198436.0 ± 37908.7	232537.0 ± 52687.3	0.114
Glutathione metabolism	94274.0 ± 19296.3	111092.0 ± 26218.2	0.120
Bacterial chemotaxis	167736.0 ± 67791.1	212372.0 ± 17748.0	0.126
Steroid hormone biosynthesis	6168.0 ± 2602.7	8245.9 ± 3450.2	0.146
N-Glycan biosynthesis	16445.6 ± 3374.7	19122.9 ± 4544.0	0.152
Biosynthesis and biodegradation of secondary metabolites	13016.3 ± 4189.3	16059.6 ± 5423.4	0.177
Fatty acid metabolism	86123.4 ± 21917.6	99566.1 ± 23254.7	0.200
Lipopolysaccharide biosynthesis proteins	244060.0 ± 50777.8	278612.0 ± 65785.6	0.205
Lipopolysaccharide biosynthesis	208132.0 ± 45877.3	239146.0 ± 59046.9	0.206
Glycerolipid metabolism	121775.0 ± 31488.7	137627.0 ± 10042.0	0.277
Linoleic acid metabolism	18968.5 ± 5153.8	21354.2 ± 5274.6	0.320
Various types of N-glycan biosynthesis	167.5 ± 116.9	134.6 ± 60.9	0.440
Bacterial invasion of epithelial cells	193.5 ± 479.7	73.6 ± 194.2	0.473
Amino acid metabolism	80748.6 ± 19153.0	86929.4 ± 19366.0	0.482

KEGG = Kyoto Encyclopedia of Genes and Genomes

DDGS = Distiller’s dried grains with solubles

Diet 1 = 0% DDGS

Diet 2 = 30% DDGS

## Discussion

The inclusion of 30% DDGS in the ration reflects a typical level, relative to industry practice [3], such that not only do these data adequately test the hypothesis that feeding 30% DDGS to pigs significantly alters the colonic microbiota and metagenome, but moreover these findings are directly applicable to similar-aged commercial growing swine consuming DDGS. In the present study, the diet containing 30% DDGS (Diet 2) had nearly twice the fiber content (NDF and ADF) and roughly 20% more protein than the diet without DDGS (Diet 1).

Wilberts et al. reported the average pH of cecal and colonic content samples from pigs fed 30% DDGS for 5 weeks was significantly more alkaline relative to pigs fed 0% DDGS [6]. This significant increase in intestinal pH in pigs fed DDGS is supportive of a diet-specific alteration in the colonic microbiota which is not unexpected as even short-term dietary manipulation (5 days or less) can induce rapid reproducible alterations in the human enteric microbiome [11]. The relative rise in cecal and colonic pH associated with feeding DDGS is also consistent with a reduction in carbohydrate fermentation as carbohydrate fermentation in the colon is typically associated with higher concentrations of short-chain fatty acids and a more acidic pH [12]. Interestingly, Urriola and Stein observed a significant increase in ileal and cecal pH in pigs fed DDGS but did not detect statistically significant differences in volatile fatty acids in these same samples [13]. Unmeasured lactic acid may also contribute to these observed differences in pH.

While Bacteroidetes and Firmicutes are consistently the two most dominant phyla in metagenomic studies of the swine gut, the relative abundance of the two is somewhat variable in the literature. In the present study, Bacteroidetes was the dominant phylum detected in all samples regardless of diet group and often accounted for more than 50% of the relative bacterial abundance. This is consistent with a recent study of adult and piglet manure where Bacteroidetes predominated in all samples regardless of age [14] but in contrast to a recent study of Chinese pigs where Firmicutes accounted for more than 75% of the detected bacterial population in the colonic contents [15]. One major difference between the present study and the contrasting study is the age of pigs when the colonic content samples were obtained. In the present study, samples were obtained from 9-week-old pigs whereas colonic content samples in the study by Zhao et al. were from 6-month-old pigs. This age difference likely accounts for at least some of the variability between these two studies as a longitudinal analysis of age-related changes in the pig fecal microbiota revealed a marked reduction in the relative abundance of Bacteroidetes in swine feces from over 30% at 10 weeks of age to less than 10% at 22 weeks of age [16]. Another recent report identified Firmicutes as the dominant phylum in pig feces regardless of age [17]; however, as these were antemortem fecal samples from pigs a direct comparison to the present study of colonic contents cannot be made. Other potential reasons for these observed differences in relative abundance of bacterial phyla between studies include differences in animal genetics, variations in diet formulations, and environmental factors. Such potential for variability further underscores the importance of consistency of methods and the inherent difficulties in making comparisons across metagenomic studies.

Consistent with the *a priori* hypothesis, feeding increased insoluble dietary fiber through the addition of 30% DDGS significantly altered the colonic microbiota, and pigs within each diet group developed similar microbial profiles. Pigs consuming no DDGS (Diet 1) had higher Firmicutes:Bacteroidetes ratios which is intriguing as higher ratios have been associated with the presence of diarrhea regardless of cause in dogs [18] and humans [19] and higher ratios were observed in pigs with “*Brachyspira hampsonii”* infection [20]. While finding lower ratios in pigs fed DDGS may suggest such a diet might have a protective effect against development of diarrhea, pigs fed DDGS were actually more susceptible to the development of swine dysentery following experimental infection [6]. Therefore while phylum-level shifts are easy to detect and may reveal trends and relative risk factors, the specific genera comprising these shifts likely drive the observed differences in disease manifestation and warrant deeper investigation. Accordingly, analysis tools such as LEfSe that incorporate an estimate of biological effect size are useful for identifying potential biomarkers within complex metagenomic data that may fail to be detected using traditional statistical tools due to the sheer volume of data.

In the present study, *Prevotella* spp. was the most abundant genus in both diet groups which is consistent with a previously published report from 10-week-old pigs [16]. *Prevotella* spp. accounted for more than 50% of the detected reads in pigs fed DDGS and this was the most discriminant feature of the Diet 2 group. This finding is also consistent with a recent human study where a positive correlation was observed between fiber intake and *Prevotella* levels in the gut microbiota samples [11] and a second study where an increased relative abundance of *Prevotella* characterized the fecal microbiome of participants with long term high fiber diets versus those with high protein and animal fat diets [21]. *Lactobacillus* spp. were significantly more abundant in pigs fed Diet 1 and were a differential feature of this diet group. This is not unexpected given the higher level of fermentable carbohydrates in Diet 1 that are likely reaching the proximal colon for microbial digestion. Considering that *Lactobacillus reuteri* is a common probiotic that has been associated with regulation of the immune system, prevention of diarrhea, and improved pig health [22], such a microbial profile might be expected to favor gut health. Additionally, *Megasphaera* spp. were a differential feature in the microbiota of the Diet 1 pigs and it has been shown that bacteria of this genus not only utilize lactate, which is produced during carbohydrate fermentation, but also stimulate production of butyrate [23], a primary energy source for colonocytes with roles in immune modulation and intestinal barrier regulation [24]. The identification of *Megasphaera* as a differential feature of the microbiota of pigs fed Diet 1 further suggests that feeding such a diet may be more favorable to overall colonic health relative to a diet containing DDGS.

The predicted gene counts from the PICRUSt analysis suggest that the colonic microbiome from pigs fed 30% DDGS (Diet 2) contains bacteria with greater capacity for protein digestion, carbohydrate metabolism, lipid and fatty acid biosynthesis, carotenoid biosynthesis, and degradation of glycans. The identification of increased gene counts for protein digestion in pigs fed Diet 2 is not unexpected given that this diet contained roughly 20% more protein than Diet 1. Increased protein fermentation in the pig colon has been associated with the formation of numerous potentially toxic metabolites such as ammonia, volatile phenols, and amines [25] which can negatively impact colonic health. Furthermore, multiple studies have shown that reducing daily protein intake can not only reduce the formation of these toxic products in the pig gut, but the incidence of post-weaning diarrhea as well [26].

Mucins, which are O-linked glycans, are the main component of mucus which has important intestinal barrier functions and is highly sulfated in the colon [27]. Loss of sulfate residues on mucins constitutes a first step in mucin degradation and there are multiple sulfatases associated with the KEGG pathways for sphingolipid metabolism and glycosaminoglycan degradation, both of which were detected with significantly greater frequency in the predicted microbiome of pigs fed Diet 2. Additionally, *Prevotella* was the most discriminant feature of the microbiota of Diet 2 pigs and it has been previously demonstrated that this organism contains a glycosulphatase with activity on mucus glycoproteins [28]. Taken together these features suggest that the microbiome of pigs fed 30% DDGS has the capacity to compromise the integrity of the mucus barrier and may in part explain why pigs fed 30% DDGS developed swine dysentery nearly twice as fast as those not consuming DDGS when inoculated with *Brachyspira hyodysenteriae* in a previous study [6]. Furthermore, recent analysis of the genome of *B*. *hyodysenteriae* revealed pathways for transport and utilization of multiple sugars (sucrose, fructose, and glucose) and amino acids [29] which should be readily available in the colonic content of pigs fed 30% DDGS based upon the PICRUSt analysis revealing increased prediction of genes associated with carbohydrate metabolism and protein digestion in the microbiome of pigs fed Diet 2.

In summary, feeding increased insoluble dietary fiber and protein through the addition of 30% DDGS induced significant alterations in the colonic microbiota with a consistent reduction in the Firmicutes:Bacteroidetes ratio coinciding with a reduction in *Lactobacillus* spp. and a predominance of *Prevotella* spp. Predicted gene counts in the observed microbial profiles from pigs fed DDGS reveal the capacity for mucin degradation and the formation of toxic end products of protein metabolism which may affect intestinal barrier function and predispose to colitis; however, as these data are based on in silico predictions experimental validation is warranted. Further investigation of microbial profiles of pigs fed increased insoluble dietary fiber in the face of enteric health challenges, such as *Salmonella* spp., *Brachyspira* spp., and *Lawsonia intracellularis* infections, appears warranted and may reveal potential biomarkers of disease susceptibility and resistance to infection.
